# The future of biofilm research – Report on the ‘2019 Biofilm Bash’

**DOI:** 10.1016/j.bioflm.2019.100012

**Published:** 2019-12-24

**Authors:** Tom Coenye, Birthe Kjellerup, Paul Stoodley, Thomas Bjarnsholt, the 2019 Biofilm Bash Participants

**Affiliations:** a Laboratory of Pharmaceutical Microbiology, Ghent University, Ghent, Belgium; b ESCMID Study Group on Biofilms, Basel, Switzerland; c Department of Civil and Environmental Engineering, University of Maryland, College Park, MD, USA; d Fischell Department of Bioengineering, University of Maryland, College Park, MD, USA; e Department of Microbial Infection and Immunity, The Ohio State University, Columbus, OH, USA; f Department of Orthopaedics, The Ohio State University, Columbus, OH, USA; g National Biofilms Innovation Centre (NBIC), UK; h National Centre for Advanced Tribology at Southampton, University of Southampton, Southampton, UK; i Costerton Biofilm Center, University of Copenhagen, Copenhagen, Denmark; j Department of Microbiology, Copenhagen University Hospital, Copenhagen, Denmark

## Abstract

In May 2019, 29 scientists with expertise in various subdisciplines of biofilm research got together in Leavenworth (WA, USA) at an event designated as the ‘2019 Biofilm Bash’. The goal of this informal two-day meeting was first to identify gaps in our knowledge, and then to come up with ways how the biofilm community can fill these gaps. The meeting was organized around six questions that covered the most important items brought forward by the organizers and participants. The outcome of these discussions is summarized in the present paper. We are aware that these views represent a small subset of our field, and that inevitably we will have inadvertently overlooked important developing research areas and ideas. We are nevertheless hopeful that this report will stimulate discussions and help create new ways of how we can advance our field.

## Introduction

In October 2005 Bill Costerton invited a small group of established and junior biofilm researchers to a ‘Biofilm Bash’ in Los Angeles (CA, USA) to discuss the rapid advances that had developed in the field over the preceding decade, to think about how new and exciting technologies could be applied to biofilm research and to identify directions that the field might develop in the future. A decade later in 2015 discussions started about having a second ‘Biofilm Bash’ to again take stock of the field and discuss both old and new challenges as well as new opportunities in biofilm research and education; this second ‘Biofilm Bash’ materialized in 2019.

From May 7 to 9, 2019, the Ponderosa Lodge in Leavenworth (WA, USA) was the scene of this ‘2019 Biofilm Bash’, organized by the late Mark Shirtliff, Paul Stoodley, Birthe Kjellerup, Tom Coenye and Thomas Bjarnsholt. The main goals were to identify the gaps in our biofilm knowledge, identify ways how we can fill those gaps, and determine how we can increase our impact related to biofilms with various stakeholders. The ‘Biofilm Bash’ was organized as a result of informal discussions between the organizers (and others) at major scientific meetings in the preceding months and years. The underlying thought that brought about the ‘Biofilm Bash’ was that the biofilm community is scattered as a subdiscipline within many other fields. For example, dental plaque, activated sludge, oil field souring, native valve endocarditis, respiratory tract infections in cystic fibrosis, microbial corrosion, indwelling medical device infections, microbial fuel cells, endophthalmitis, osteomyelitis, and prosthetic joint infections are all biofilm-related – i.e. the mechanisms behind them have many commonalities, yet we lack the unity that can be found in other research topics (e.g. cancer).

**Fig. 1:**
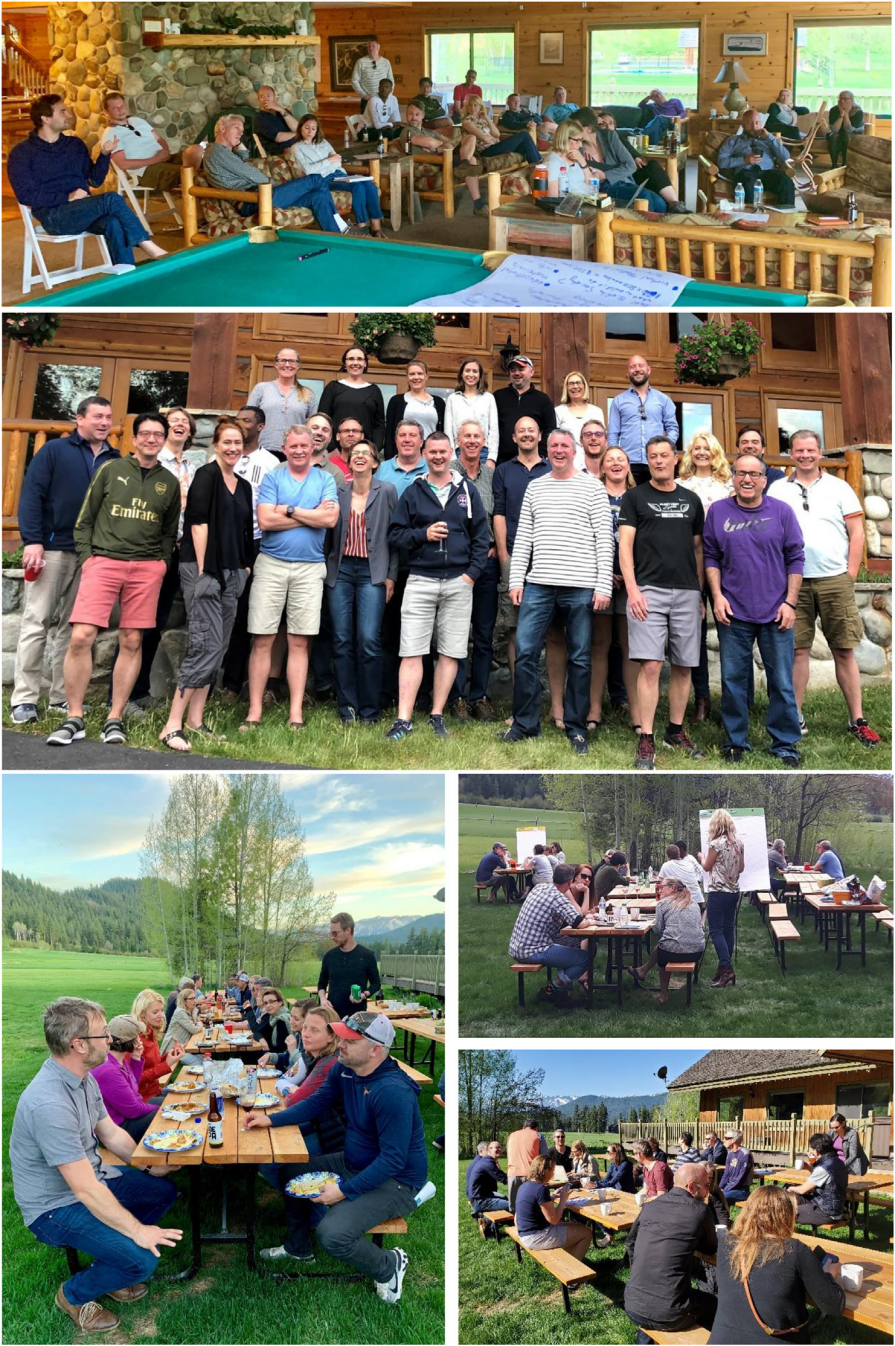
**Selection of pictures taken during the 2019 Biofilm Bash.** Top: plenary session inside the Ponderosa Lodge. Middle: Group picture with all participants in front of Beaver Creek Lodge. Bottom left: Dinner on the first night. Bottom right: discussions on the meadow.

An integral part of the meeting preparation included invitation of participants that would reflect the overall diversity of the biofilm field and at the same time created a balance between more established biofilm researchers and people that are new to the field and/or come in from a different angle. To allow fruitful participation, manageable and productive small group discussions (with max. six people in each group), and because of logistical reasons, the target number of attendees was set at approx. 30. Potential participants were identified in a decentralized way, where Birthe Kjellerup, Darla Goeres, Kendra Rumbaugh, Matthew Parsek, Paul Stoodley, Thomas Bjarnsholt, Tom Coenye, and Trine Rolighed Thomsen (all involved in an informal ‘pre-meeting’ on 9 October 2018, in Washington DC, during the American Society for Microbiology (ASM) Biofilm Conference) were asked to each propose up to six names, and all these people were pre-invited. Ultimately 29 researchers from Portugal, USA, Denmark, Belgium, The Netherlands, UK, and Australia were present (Table 1, Fig. 1).

**Table 1:** Overview of participants and their affiliation.

Name	Affiliation	City, country
Nuno Azevedo	University of Porto	Porto, Portugal
Haluk Beyenal	Washington State University	Pullman, WA, USA
Thomas Bjarnsholt	Costerton Biofilm Center	Copenhagen, Denmark
Mette Burmølle	University of Copenhagen	Copenhagen, Denmark
Tom Coenye	Ghent University	Ghent, Belgium
Vaughn Cooper	University of Pittsburgh	Pittsburgh, PA, USA
Matthew Fields	Center for Biofilm Engineering	Bozeman, MT, USA
Darla Goeres	Center for Biofilm Engineering	Bozeman, MT, USA
Luanne Hall-Stoodley	The Ohio State University	Columbus, OH, USA
Birthe V. Kjellerup	University of Maryland	College Park, MD, USA
Michel Koo	University of Pennsylvania	Philadelphia, PA, USA
Kasper Kragh	Costerton Biofilm Center	Copenhagen, Denmark
Bastiaan Krom	Academic Centre for Dentistry	Amsterdam, The Netherlands
Tagbo Niepa	University of Pittsburgh	Pittsburgh, PA, USA
Matthew Parsek	University of Washington	Seattle, WA, USA
Gordon Ramage	University of Glasgow	Glasgow, UK
Courtney Reichhardt	University of Washington	Seattle, WA, USA
Alex Rickard	University of Michigan	Ann Arbor, MI, USA
Katharina Richter	University of Adelaide	Adelaide, Australia
Trine Rolighed Thomsen	Aalborg University and Danish Technological Institute	Aalborg and Aarhus, Denmark
Kendra Rumbaugh	Texas Tech University Health Sciences Center	Lubbock, TX, USA
Karin Sauer	Binghamton University	Binghamton, NY, USA
Pradeep Singh	University of Washington	Seattle, WA, USA
Phil Stewart	Center for Biofilm Engineering	Bozeman, MT, USA
Paul Stoodley	The Ohio State University, and National Biofilms Innovation Centre (NBIC), University of Southampton	Columbus, OH, USA
Jeremy Webb	National Biofilms Innovation Centre (NBIC), University of Southampton	Southampton, UK
Marvin Whiteley	Georgia Institute of Technology	Atlanta, GA, USA
Craig Williams	University of West Scotland	Paisley, UK
Dan Wozniak	The Ohio State University	Columbus, OH, USA

To allow maximal input and involvement from all participants, the ‘2019 Biofilm Bash’ was organized as a series of small-group brainstorming sessions during which six questions were addressed. There were no research presentations. These six questions were proposed by the organizing committee but were fine-tuned based on the input from the majority of participants. Each question/topic was discussed by at least four groups of four to six participants. In addition, to maintain the dynamics, groups were re-organized after every session to mix participants. In every group a person was designated as discussion leader; this discussion leader was also responsible for reporting back to the entire group during two plenary sessions. The outcome of these discussions is summarized below.

Question 1 **What will be the technologies of the future in biofilm research? Which are the developing technologies in this field and what technologies from outside the biofilm field could have a major added value? How can we ensure that methods commonly used in environmental research find their way to clinical research and vice versa?**

The three main themes that emerged were the need to incorporate techniques which can a) combine microscopic imaging with label free chemical profiling, b) sense the biofilm environment and c) process large and complex data sets (such as omics data) with machine learning and artificial intelligence to reveal patterns in multi-scalar relationships and interactions between the biofilm and its environment.

**Biofilm imaging.** Imaging has been the mainstay of biofilm research and provided the first direct proof of these communities on surfaces in the early 1980s [1,2]. Initially scanning electron microscopy (SEM) was used to provide high resolution images of the surfaces and transmission electron microscopy (TEM) was required to resolve the ultrastructure between the cells and the EPS (extracellular polymeric substance) but these techniques require dehydration and labelling of specific components is difficult. Biofilm imaging was revolutionized in the early 1990’s by confocal microscopy which allowed live cell imaging in 3D in real time [3,4]. However for most purposes labelling is limited to a few compounds and the visualization of individual EPS polymers and other components such as vesicles and determining how they structurally interact among themselves and with the bacterial cells is beyond the limit of resolution of this technology. Super high resolution (beyond the diffraction limit of light) live cell imaging is now becoming increasingly available with resolutions from 100 ​nm to 20 ​nm (in the XY plane), which will allow us to probe these interactions [5]. Another exciting area is that of label free imaging techniques such as Coherent Anti-Stokes Raman Spectroscopy (CARS), which can be coupled with confocal microscopy or matrix-assisted laser desorption ionization mass spectrometry (MALDI-MS) and other MS imaging techniques to provide high resolution chemical mapping of a specimen [6]. Imaging the EPS has been difficult not only due to limitations in microscope resolution but also because there are no universal stains and so only targeted components can be detected. Chemical imaging has great potential to investigate EPS chemistry and can also be used to identify components such as antibiotics or signals within biofilms. There was some discussion on the need for more sophisticated image analysis techniques for quantifying biofilms, which are less subjective than those used today. They largely rely on semi-subjective thresholding and are also limited by the stains and concentrations that the microscope can detect [7].

**Sensors.** Monitoring heterogeneity in the biofilm microenvironment can sometimes be achieved with specific stains. However, in many cases, particularly in the presence of dissolved components such as nutrients or metabolites, there are no stains available or the time scale is too slow. In addition, direct microscopic examination might not be feasible outside the lab (i.e. *in situ* such as in a wound or an industrial pipeline). Currently microelectrodes and planar optodes can be used but these can be fiddly and require direct access and sophisticated equipment thus they are most used in the controlled environment of the laboratory. Nanobots and nanorobotics is an area that is being developed in the field of medicine to send wireless signals to report on the local environment [8]. It was discussed that such technology would have great application in basic and applied biofilm research.

**Artificial intelligence (AI)/machine learning (ML).** We are currently living in the age of ‘omics’, and although various ‘omics’ approaches are being applied to biofilm communities, relating these large and usually complex data sets to biofilm function still often relies on manually cherry picking a handful of familiar or likely candidate genes or proteins, or using principal component analysis to differentiate between overall patterns. We are still playing catch-up with providing the infrastructure for interpreting such data sets and bioinformaticians are still in high demand. It was predicted that AI/ML will develop in a similar way and offer great potential in recognizing complex patterns in biofilm data sets (see for example reference [9]), integrating disparate data sets such as 4D imaging data with multidimensional chemical data and ‘omics’ data sets.

**Others.** In addition, many other biofilm related techniques were brought up including mechanical and rheological testing, flow cytometry, cryo-EM and more sophisticated computational modeling software for integrating biofilm processes with fluids interactions. Some techniques such as microfluidic platforms for high throughput experiments and the integration of multi-scalar correlative imaging techniques, i.e. Confocal Raman Microspectroscopy (alone or combined with stable isotopic probing) [10], live cell imaging, small animal imaging with *in vivo* imaging systems (IVIS) [11] and medical imaging techniques such as Micro Computed Tomography (micro-CT) [12] or Magnetic Resonance Imaging (MRI) [13] with resolutions ranging from the sub-cellular level to the system level, are clearly promising but were not discussed in detail. In the medical field, medical imaging techniques which could show the location of biofilm pre- or intra-operatively would present a major breakthrough for surgical management as well as allowing the progress of treatments to be tracked as they are for tumor treatments. Currently there are no biofilm specific probes and the spatial resolution of medical imaging modalities is not sufficient.

Question 2 **What innovative approaches to tackle unwanted biofilms are needed? What can be expected from them?**

The discussion focused primarily on **non-antibiotic or antibiotic adjuvant prevention and treatment strategies**. These included targeting the EPS matrix to break up the biofilm either enzymatically or through activating natural dispersion mechanisms [14], adding ‘potentiators’ such as metabolites or nutrients which may activate persister and stationary cells, rendering them susceptible to antibiotics [15,16], and vaccines against biofilm specific antigens [17]. Based on developments in tumor cancer medicine, a suggestion was to use immunomodulation therapy to disrupt the pathogenic microenvironment. It was suggested that a solution for prevention was a surface that was inherently repulsive to bacteria, yet was biocompatible in the clinical setting and mechanically functional in engineered systems. However, as bacteria are not always attached to the surface of the implant but maybe present as aggregates close to the surface, this may not be the perfect solution and more studies are needed to address this. There are interesting developments in biomimicry approaches based largely on physical patterning [18], although these tend to be expensive and there are questions on whether patterning alone can fully explain the biological function.

In addition to discussing novel therapies it was recognized that **we are still lacking a basic understanding of how antibiotics interact with biofilms**. It is well established that cells in biofilms are orders of magnitude less sensitive to antibiotics and antimicrobial agents than rapidly growing planktonic and early solid agar plate cultures. These data are largely based on 24 ​h exposure times or less. For medical biofilms anecdotal clinical evidence suggests that biofilms can survive long term exposure to systemic antibiotics but in this case the concentration is limited by the threshold of the therapeutic window, beyond which toxicity is a concern. Basic research is needed to define the pharmacokinetics with respect to high concentrations, which might be achieved through local targeted delivery and over longer time scales. Another important factor is to determine how the age of the biofilm influences susceptibility and how translational *in vitro* tests are to the real world environment. The need for better models was discussed in more depth in Question 3 (see below).

Along the same lines, the question was asked with any therapeutic **how low is low enough**? It is not clear how many percent or log reductions in biofilm bacteria is enough, or how the absolute magnitude of numbers which might remain, relate to clinical or industrial relevance. Each system will have a specific tolerance and in some cases (such as certain infections) complete eradication is required (according to conventional thinking), whereas in some industrial systems (e.g. ship hull fouling) a limited amount of biofilm may be tolerated as long as it does not cause negative effects (e.g. drag) beyond a critical operational level. Interestingly, there was some discussion of whether the body could tolerate a certain amount of pathogenic biofilm bacteria and if therapeutics might still have utility if they keep the biofilm in check, such as is the case with routine oral hygiene or the use of suppressive antibiotic therapy in patients, where surgery is contraindicated and the infecting bacteria are susceptible to well-tolerated antibiotics.

A different approach to control medical biofilms was to use approaches to **reduce bacterial adhesion and biofilm build up** that are commonly used in the natural environment and in industry. In pipelines for example, dead ends where water stagnates, lags, sudden expansions and contractions, sharp bends and surface protrusions are reduced, since it is known that turbulent eddies associated with these features can trap and promote bacterial attachment to surfaces. Eddies and back current locations might furthermore form protective niches. A similar phenomenon occurs in infective endocarditis, where damage to the heart valve allows bacteria to accumulate and proliferate in biofilm vegetations [19]. Thus some of these design concepts, along with the use of materials that can be more easily cleaned, might be applied to medical devices and the hospital environment as infection control measures.

**Probiotic approaches** were also discussed. The use of probiotics is now accepted with respect to gut microbiology but increasingly there is discussion among surgeons, at least in orthopedics, that probiotics might be used to prevent or manage deep surgical site infections. A few years ago discussion of introducing bacteria to a deep tissue site would have been unthinkable; now there is discussion of native joint microbiomes [20,21] and the role of a healthy lung microbiome in infectious diseases [22,23], opening up the door to thinking about novel therapeutics.

Question 3 **How good are our** **experimental** **models? There is no framework for biofilm model evaluation in regard to how well it recapitulates the natural environment it is meant to mimic. How can we approach this question as a field?**

Models are essential to science and a variety of models are used in biofilm science ranging from simple *in vitro* models to more complex invertebrate and vertebrate *in vivo* models [24,25]. The discussion showed that there is a **lack of standardization** for experimental protocols and data analysis (see also below, Question 5). A publicly accessible database hosting currently-used methods with protocols and standards was proposed. This would be followed up by workshops at general and more specific conferences and meetings during, which (starting) biofilm researchers could be trained in these methods. The workshops would utilize material from the database and over time this repository could become interactive. To make it easier to perform comparable experiments with selected models, it was suggested that lists of preferred vendors would also be present in the database.

An important part of the discussion was related to “**How do you select an appropriate model?**” It was suggested to develop a Decision Tree for model selection that would include criteria for choice of models as well as parameters that should be discussed during the experimental phase and subsequently for data analyses. This would make it possible to compare and evaluate results from similar models but from different research groups. It was also suggested to develop a list of parameters that should be included in manuscripts and proposals to ensure a more informed and fair evaluation. It was discussed what would be sufficient and what will be necessary if guidelines would be developed and enforced. When is a simple model sufficient to answer the specific research question, when should one move to a more complex model and which types of parameters should be evaluated at each step? It was also discussed how it can be ensured that researchers in more resource limited settings (such as many undergraduate colleges and/or less resource rich countries) could still be included if such guidelines were implemented. An important aspect of model selection is to ask the right question before making a decision about a model. In this consideration, the presence of spatial and physiological heterogeneity needs to be accounted for.

**The *in situ* relevance of the biofilm model must also be evaluated prior to selection,** for example, prior to using a particular model, it should be evaluated whether the inoculum, the flow regime (if any) and the surfaces/interfaces are matching the environment in the system that is being modelled. The key drivers and parameters in the system and environment must be identified to obtain *in situ* relevance. Tools should be developed that can be used for evaluation of how the model and results can be transferred to practice and the practitioners (including clinicians and engineers) should be involved in evaluation of the model to ensure *in situ* relevance. Subsequently validation studies should be performed (e.g. can a toilet bowl as a system be modelled in a Rotating Disk Biofilm Reactor?). The host response is also an important factor for consideration of medical/clinical biofilm models. An alternative to conventional vertebrate models (mouse, rat, rabbit) are zebrafish, and invertebrate models such as *Galleria mellonella* or *Caenorhabditis elegans* in cases where only the innate immune response is investigated [26]. Organ-on-a-chip options are increasingly being applied and benefits from the opportunities of 3D-printing and micro-hydraulic systems [27].

A discussion point was raised that **more knowledge is needed about the environment that is being investigated prior to the selection of a model**, since the environment often determines the organization of a biofilm. This will enhance knowledge about complexity while simultaneously dealing with reproducibility and the pragmatic issue of finances for repeated experiments. Also, while bad models do not exist, a particular model can be poorly suited for answering a particular research question and/or the interpretation of the obtained data can be bad. Appropriate quality assurance (QA)/quality control (QC) procedures must be in place as with all experiments and must include the quality of the microbial inoculum as well as the rest of the model system. This will ensure a higher degree of reproducibility. The details of the applied methods must be outlined as a part of the QA/QC. The need to develop relevant models for polymicrobial interactions such as infections as well as environmental systems and many industrial problems was raised. Stable and maybe even self-sustaining model systems are also needed. The complexity of existing *in vitro* and *in vivo* models was discussed. Can we -based on existing data-evaluate how good these models are? Better approaches for evaluation of biofilm models must be developed so they accurately recapitulate the *in vivo* setting. Is it necessary to apply transcriptomic, proteomic and/or phylogenetic techniques to document that the applied model is accurate and can this be utilized in complex environments? Biofilm communities per definition are complex and heterogeneous and we need to ensure that the complexity is modelled appropriately. However, it is currently unclear whether all systems can be modelled. Computer modeling can potentially solve some of the gaps that arise from experimental approaches.

Finally, there was interest in identifying a medium, where **negative data** can be published, since these are often showing the limitations and negative sides of models that are otherwise neglected.

Question 4 **How can we make other stakeholders (e.g. medical doctors, engineers, other professionals) aware that biofilms are important? What can we do to make sure that the biofilm concept is actually included in curricula and standardized tests? Can something like the biofilm ‘hypertextbook’ developed at the Center for Biofilm Engineering (CBE) play a role in this? What about developing workshops that can ‘travel’** to **the different biofilm conferences and be renewed as needed?**

**Increased visibility** to ‘biofilm’ as an integrated part of other scientific fields was requested. This can be done by publishing reviews in relevant journals as well as by interacting with the lay press (e.g. informing newspapers and local media about new discoveries and overall information about biofilms). Increased use of social media as a platform should also be considered to reach younger demographics. Informative YouTube videos and TED-style talks by high-profile scientists and other thought leaders could also be a part of ‘marketing’ the biofilm concept. We can increase the visibility of ‘biofilm’ by always including it as a keyword, use social media (Twitter, ResearchGate, LinkedIn etc.) to highlight biofilm-related publications but also by writing position papers to be published in journals that typically do not have a biofilm focus. Publication of case reports involving biofilms will increase the awareness about biofilms in the clinical and industrial fields and can be delivered in a way that will promote appreciation of biofilms when they occur in the these settings. Furthermore, a correction of decades of misconception in the field that a biofilm is a surface-attached mushroom-shaped structure, must take place. This will likely increase the general awareness, since people will be able to associate and identify their findings as a biofilm even though it is not a mushroom-like structure. For the biofilm message to be received, the message must be defined and refined so it will hit the target audience. The American Cancer Society has been very successful in delivering their message (supported by effective fundraising) and it was suggested that this could serve as a model. In this way biofilms can be linked to important societal issues such as antimicrobial resistance, microbial corrosion and chronic infectious diseases, but also to the positive aspects related to for instance wastewater treatment, food production and bioremediation. An important aspect for this part to move forward is to identify existing data on the burden and benefits of biofilms, respectively. It is also imperative to link biofilm to other recognized concepts such as cancer, global warming (including coral bleaching), and food production, and to estimate the financial and human global burden.

**Primary and secondary education (K-12 in the US) as well as education at the university level** are important gateways to increasing the awareness and knowledge about biofilms. More focus on providing information and materials for teachers at all levels will increase the general awareness. This could be done by providing examples of harmful and beneficial biofilms for different levels that educators can include directly in their lesson plans and in the classroom. Experimental biofilm instruction can also be facilitated by examples and protocols that high school and university students can benefit from. Establishment of a database with protocols and vendor information (cfr. Question 3) would also be a part of improved outreach to educators. Wikipedia and other links could add to the knowledgebase for middle and high school students. Resources already exist such as ‘Evolving STEM’ (https://evolvingstem.org/) that can be included right away.

**Continuing education of professionals** at major conferences and other venues will also be an important aspect. Several medical organizations offer courses, workshops etc. to obtain CME (Continuing Medical Education) and CE (Continuing Education) credits. The topic of biofilm should become a part of CME/CE and other types of continued education for other organizations. To get biofilm to be a part of CME/CE, increased impact of biofilm must be pushed at the general microbiology meetings that take place at an annual basis such as ASM Microbe, the Federation of European Microbiological Societies (FEMS) Congress of European Microbiology, The Microbiology Society Annual Meeting (UK) and the European Congress of Clinical Microbiology & Infectious Diseases (ECCMID). This could also provide an avenue for making ‘biofilm’ a topic for the Medical Board Exam, Dental Board Exam, and the Fundamentals of Engineering Exam (the Engineering Board exam). The push for inclusion on the Board Exams has a straightforward path in the US. However, in Europe and globally, this must be individually targeted for each country.

Question 5 **Standardization/databases/data sharing. Should there be a biofilm database in which large datasets relevant to the field are uploaded to prevent the need for each group to scour existing databases. If so, where should it be maintained and who can curate it? Maybe crowdsource? This is an issue for labs that don’t have the expertise to analyze large datasets and for labs that focus on large datasets.**

**Should we standardize our models? When is standardization required?** The question about ‘standardization’ is not new (see also Question 3, above), but remains relevant in the biofilm research field and is frequently the subject of debate. Standardization of methods is often driven by regulatory authorities (e.g. the US Food and Drug Administration [FDA], the US Environmental Protection Agency [EPA], and the European Medicines Agency [EMA]) and industry, and this is also the case in the biofilm field [[28], [29], [30], [31], [32]]. Standard methods allow for comparison across treatments and devices, enable regulatory agencies to make informed decisions, and provide insights into the Type 1 and Type 2 error rates that can be expected, when the treatment or device is tested in a clinical or industrial trial. Standard methods used for applications beyond their intended ‘significance and use’ may provide misleading results, but the error is the result of misuse of the method, not due to the inadequacies of the standardization. Finally, standard methods are living documents that require constant review and updating. As science and technology evolve, so should the standard methods. Standardized methods can also play an important role in increasing our understanding of the basic biology of biofilms, including insights into the mechanisms involved in biofilm tolerance. Comparison of results obtained in absence of some form of standardization is at best difficult [33]. Standardized methods are important tools for screening of large libraries for potential compounds with anti-biofilm activity [32]. However, regardless of standardization, *in vitro* biofilm susceptibility tests will frequently (but not always, [[34], [35], [36]]) yield results that are poorly representative of the actual activity of the antibiotic against the biofilm *in vivo*, because of profound difference between different types of biofilms. This is an important point, because it illustrates that even the best *in vitro* model does not fully mimic the *in vivo* situation. Progress has been made in developing laboratory biofilm models that are more representative of the situation in a patient (e.g. in the context of chronic wound infections and CF) [37,38] and industrial environments [39], but studies establishing the validity of these models are still largely lacking. Finally, in some contexts it may be more readily achievable and useful to standardize specific key parts of workflows and procedures, rather than the entire method. For example, it may be difficult to standardize the entire workflow of biofilm formation, antimicrobial treatment and crystal violet staining in a 96-well microtiter plate, but the staining procedure as such could be standardized.

**Do we need biofilm-specific repositories? What about sharing and re-use of data?** Standardization would also improve the content of databases, and the other way around, databases can promote the standardization efforts. Databases in the context of biofilm research could refer to physical databases for microbial strains, and to computer databases for a wide range of different data types (images, genomics, transcriptomics etc.).

For the deposit of (mutant) strains no additional initiatives are necessary. Strains can be deposited in a number of international culture collections (including ATCC in the US and BCCM/LMG Bacteria Collection in Belgium). This deposit guarantees that a particular strain is not lost and allows other researchers to easily obtain it. In addition, special panels of microorganisms can be assembled and deposited in these collections. A recent example of this includes the international *Pseudomonas aeruginosa* reference panel [40]. The deposition of (sets of) strains in international culture collection will allow researchers from different laboratories to work on the same strain, which will facilitate comparison of results obtained with different experimental approaches in different laboratories. Making sure that biological material included in published biofilm studies is available to other researchers is a shared responsibility of authors and journals/publishers; with the need for the latter to be stricter about this.

There is less consensus about the deposition of data, although overall the feeling was that one or more repositories dedicated to biofilm data could be useful. As an example, the WormBase repository for data concerning *Caenorhabditis elegans* and related organisms was mentioned [41]. However, the added value of depositing biofilm-derived ‘omics’ data in a second database (besides e.g. GenBank and ArrayExpress) was questioned. The experience with the BiofOmics web platform aimed at the systematic and large-scale compilation, processing and analysis of biofilm data from high-throughput experiments [31] shows that it is difficult to convince researchers to deposit their data and the curation of such database(s) is time-consuming. A possible solution to that is crowdsourcing of curation, but that solution carries the risk of introducing bias and variability. In addition, the main goal of data deposition is re-use of data and this has several implications. Besides the issue of standardization outlined above, there is also the issue concerning metadata – i.e. what information about a dataset is required before data can be re-used? In 2014, standards for reporting experiments and data on biofilms were published (minimum information about a biofilm experiment, MIABiE) [30] but the uptake of these guidelines has been slow, both on the side of the scientific community (i.e. biofilm researchers) and on the side of journals/publishers. Regardless, there seems to be a consensus that more efforts are needed to make raw data and the associated relevant metadata available as soon as possible, at the latest when the research results are published. This is a shared responsibility of authors and editors. A final added value of a biofilm data repository is that it would force researchers to upload their data and metadata in a certain way, opening up the possibilities of performing meta-analyses on these data.

**Can we provide guidance to (starting) biofilm researchers?** With the ever-growing number of biofilm-related papers published and the wide range of different methods available, it is not easy for researchers new in the field (or new to a particular subdiscipline in the field) to choose the most appropriate model system (see also Question 3). While there are several review papers on various aspects of biofilm model systems and biofilm methods in general [24,25,38,42], it seems that this information is not easily available for researchers. An example of this is the widespread use of crystal violet staining. While this can be a valuable tool, it is often used in experiments that would be better served by other output parameters. In addition, some guidance towards data interpretation may also be needed (e.g. how much biofilm reduction is needed in a particular model before considering it biologically meaningful?). A comprehensive decision making algorithm (cfr. Decision Tree for model selection in Question 3, above) for choosing the most optimal approach for a particular biofilm experiment would be considered useful and detailed information about biofilm methods (principles, protocols, examples of studies using these protocols) should ideally be available online, e.g. as part of the ‘Biofilms: The Hypertextbook’ website (http://www.hypertextbookshop.com/biofilmbook/v004/r003/) maintained by the Center for Biofilm Engineering.

Question 6 **What do we see in the future of the field in terms of journals, conferences, organization, training schools? Is there a way to (re-)unite the field in terms of conferences - is this necessary or are we happy the way it is? What model(s) do we see in the future for biofilm research? Is there need for more and/or larger (virtual) centers like** **the** **CBE (Center for Biofilm Engineering), NBIC (National Biofilms Innovation Centre) and SCELSE (Singapore Centre for Environmental Life Sciences Engineering)? Is there a need for an independent biofilm society focusing on biofilms in health, environment and energy?**

Although biofilms impact all aspects of life and biological sciences, it is viewed as a separate field. Is this due to historical reasons, with bacteriology historically involving shaken cultures and mostly planktonic bacteria or is it because we as a field have isolated ourselves on purpose (e.g. by organizing dedicated biofilm conferences)? Biofilms are likely involved in most (if not all) aspects of microbiology, but this is often overlooked.

**Organization within the field and outreach.** One of the key points of the ‘Biofilm Bash’ was to discuss whether an International Biofilm Society should or could be formed. This was welcomed as an intriguing idea, and would give visibility to the field, but there were also many questions about its goals, the practical organization and financial aspects. Several societies for microbiology already exist and it was argued that forming an International Biofilm Society would tap into the energy and passion that people have for the field and may dilute and financially damage the existing societies. The general feeling was that it would be better to use the existing microbiology societies (including ASM, FEMS and the European Society for Clinical Microbiology and Infectious Diseases [ESCMID]) to ‘push the biofilm agenda’ while at the same time opening up the field to others (including bioengineers, mathematicians etc.). Successful global outreach and public engagement will likely require more educational material, position papers, and text books (see also Question 4). A list of future stakeholders include -but is not limited too-schools, universities, industry, regulatory agencies and governments.

**Conferences.** Another point for discussion was biofilm conferences: do we need more or less? There was a general consensus that we should not have more conferences on biofilms, the field is saturated. Rather we should aim to have more joint meetings as too many meetings dilute the impact and attendance. A preferred scenario is to have one biofilm conference every year with large joint conferences every 3rd or 6th year. It has been proposed before to combine the different biofilms conferences but so far attempts have been unsuccessful. However maybe in the light of the ‘Biofilms Bash’, we could approach the different biofilm conference organizers to discuss a united approach. The consensus was that we need to rely more on existing microbiological societies and their (general) meetings to organize these conferences. It was also proposed to setup a Gordon or Keystone conference on biofilms. Strategies for future meetings should be directed towards reaching people who are new to the field: we need people to understand that biofilms are not a special thing but rather the common mode of growth of microorganisms!

**Journals.** As for biofilm specific journals, it was thought that the market is already saturated. With the new journal ‘Biofilm’ we have enough specific journals and biofilm research can also be published in the existing more general journals.

**Training schools and courses.** It was discussed how we can educate researchers about biofilm and use existing knowledge and resources better. Training schools and workshops, such as Summer Schools, to learn the basics of biofilm models are still needed but it is worth investigating whether this can be organized at a larger international scale (Woods Hole and Cold Spring Harbor course programs were mentioned as examples) as such courses would benefit from a more interdisciplinary approach. There also seems to be a need for shorter/more dedicated courses also offering data analysis, including statistics. Other suggestions for workshops and short courses/training schools include image analysis, flow cells, microscopy, industrial biofilms, regulatory aspects of biofilms, hands-on techniques, clinical/medical biofilms, dental biofilms, environmental biofilms, and biofilms in engineering. These courses/workshops could also be part of conferences as is already happening with Eurobiofilms, ASM Conference on Biofilms and ChinaBiofilms. It is proposed to make a list of available hands-on courses as well as online courses (e.g. https://biofilmcourse.ku.dk/, https://www.coursera.org/learn/bacterial-infections).

**Networking.** The biofilm field would benefit from a map of available infrastructure, resources, societies, as well as integrative conferences and courses. Increased interdisciplinary and international networking would also be beneficial and the larger centers (e.g. NBIC, CBE, SCELSE) can and should play a role in catalyzing these interactions. This was discussed extensively and although the value of this (e.g. in leveraging international funding initiatives) was clear to all, organizing this on an international level is challenging. Funding agencies that could be contacted for this include the National Science Foundation (NSF, US), National Institutes of Health (NIH, US) and the European Cooperation in Science and Technology (COST) intergovernmental framework. Regardless of which partnerships/networks will develop in the future, these should be inclusive. We will need to act as a connected community to successfully develop platforms that fuel innovation!

## Concluding statements

It is very difficult to capture everything that was discussed between 29 people during two days of engaged and animated discussions. Nevertheless, we are convinced that the most important issues are covered in the summary above. In addition, during the plenary sessions, several action items were identified. These are often transversal (i.e. cut across the different questions) and are summarized in Box 1. With the support of the wider biofilm community, we will start addressing those action items in the near future. Progress updates will be presented at the various biofilm conferences and other meetings as well as in writing.

Box 1 **Action items identified during the plenary discussions.**

• Techniques used to study the microenvironment of solid tumor cancers and for *in situ* research of microbial communities in the natural environment have great potential for application to laboratory biofilm experiments, and should be translated to other fields such as industrial, medical or dental biofilms. One way to achieve this would be for biofilm researchers to attend methods intensive conferences outside the biofilm field. The International Society for Microbial Ecology (ISME) conference was cited as an excellent example.
• A second path would be to bring in speakers from other fields to future biofilm conferences whom were developing methods in their own fields which may be translatable.
• Workshops dedicated to specific techniques which might have biofilm applications could help advance the field.
• A better overview of what is available (worldwide) in terms of training opportunities is desirable and it needs to be explored how different initiatives can be connected.
• A well-documented study demonstrating that a biofilm specific therapeutic has clinical efficacy, where conventional surgical or antibiotic interventions had failed, would advance the medical biofilm field. If a biofilm approach could be demonstrated in one type of infection, then the doors might open to spark innovation in treating other types of biofilm infections.
• In terms of surface modifications, silver alloy in the coating of a urinary catheter has demonstrated a reduction in infections [43]. However, the mechanism of action, whether it reduced adhesion or influenced biofilm development is un known. More fundamental knowledge on the mode of action of successful surface modifications beyond silver alloys is needed.
• The lack of a definitive biofilm biomarker and medical imaging modality for detecting biofilms makes it difficult to assess the impact of anti-biofilm therapeutics. Thus work towards a suite of biofilm markers is needed.
• There is need for a position paper on the applicability and limitations of biofilm and host/environmental models. Topics such as the ones listed below could be discussed in this paper: (i) Define constraining parameters in model systems with respect to spatial and temporally relevant scales. This in an engineering approach, where the goal is to define the principal features that we need in a model given that the complexity prohibits all variables. (ii) Development of enhanced tools for accurate evaluation of the applied model. (iii) Application of computer models to aid in evaluation. (iv) Assess the utility of artificial intelligence in biofilm control evaluation.
• Wikipedia is often the first source of information (lay) people find, so we need to make sure the ‘Biofilm’ Wikipedia page is current and updated.
• To deliver a clear and consistent message about what a biofilm is, a set of slides (1–3 slides) will be developed that can be shared by all interested.
• There is an urgent need to bring a clear message to stakeholders about the importance of biofilms. No reliable estimates of the overall burden of biofilm infections have been published since 2010 [44] and an updated estimate of the human, financial, and social burden of clinical and industrial biofilms is needed. What are the benefits of biofilms in certain systems? What costs are associated with biofilm-related fouling? Different target audiences will need different clear messages. An essential aspect of this is bringing the biofilm concept to the attention of funding organizations. Antimicrobial resistance and microbiome management are now accepted areas of concern and targets for therapeutics but we need to better incorporate the concept of biofilms into these discussions to highlight issues with controlling biofilms. In parallel, the benefits of biofilms should also be estimated and delivered to stakeholders interested in promoting biofilm.
• Development of a Biofilm Text Book could help reach a wider audience. The current version hosted by the CBE could be further developed.
• We need to ‘push’ the biofilm concept with scientific and professional organizations so it is included in recommended training programs, continued education activities and curricula.
• There are several misconceptions in the field, including that biofilms always take the form of mushroom-shaped structures, that the process of biofilm formation follows a fixed developmental cycle, and that all biofilms are surface-attached. These misconceptions need to be corrected and the message delivered to a wide target audience.
• We need to discuss to what extent we expect biofilm experiments to be standardized and if so in what aspects of the workflow standardization is most important. Guidelines that reflect this should be developed. ‘Minimum information’ guidelines [30] can support researchers, reviewers and editors to determine whether the information required to successfully reproduce an experiment is available. In addition, we need to make sure these guidelines are disseminated widely to ensure that they are adopted by all stakeholders.
• There was a consensus that there is currently no need for a separate biofilm society. However, if we want to further integrate the field at an international level, we need to explore funding opportunities and talk to funding organizations and program officers.

Alt-text: Box 1

## Author contribution

Considering the special nature of this paper and the author list, we prefer not to include an *Author Contribution Statement*, as we feel that in this case this had no added value.
